# Supplementary material for: Identification of photoperiod-induced specific miRNAs in the adrenal glands of Sunite sheep (Ovis aries)
Source: Front Vet Sci. 2022 Jul 22;9:888207. doi: 10.3389/fvets.2022.888207 (PMC9354845; doi:10.3389/fvets.2022.888207)
Supplement: Supplementary file 1 [file Data_Sheet_1.ZIP › Supplementary file/miRDeep2 file.zip!/miRDeep2 file/Know_Structure/SP42A1/oar-mir-200c.pdf]

miRBase precursor : oar-mir-200c
Total read count : 198
oar-miR-200c read count : 198
remaining reads : 0

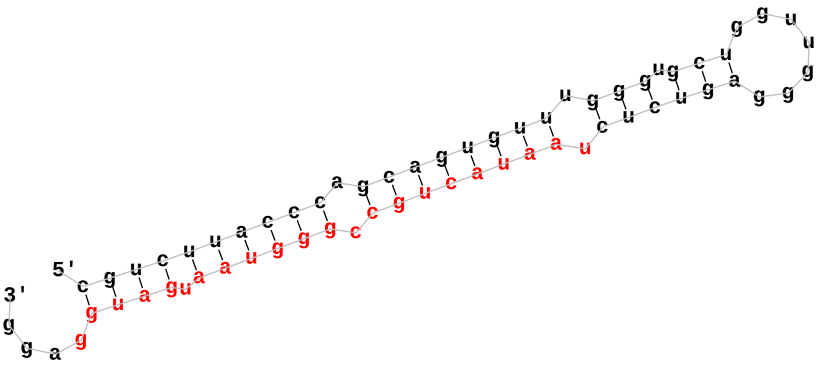

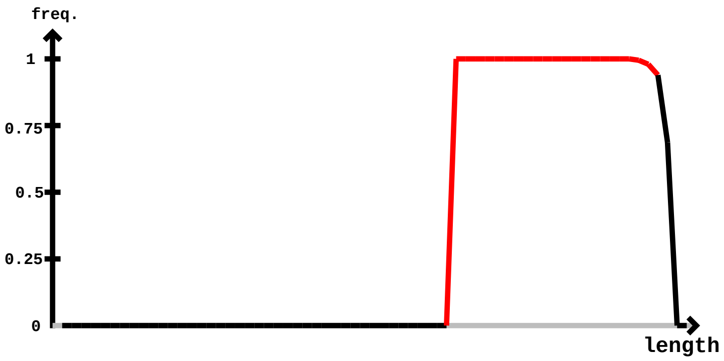


```
                                        oar-miR-200c
5'- cgucuuacccagcaguguuugggugcugguugggagucucuaauacugccggguaaugaugggagg   -3'    exp
    ((((((((((.((((((((.(((.(((.......)))))).)))))))).)))))).))))....    reads      mm         sample
    ........................................uaauacugccggguaauga......    1          0          seq
    ........................................uaauacugccggguaaugau.....    3          0          seq
    ........................................uaauacugccggguaaugaug....    8          0          seq
    ........................................uaauacugccggguaaugaugg...    50         0          seq
    ........................................uaauacugccggguaaugaugga..    136        0          seq
```
